# Symmetrical Drug-Related Intertriginous and Flexural Exanthema (SDRIFE)-Like Presentation in a Case of Systemic Contact Dermatitis to Paraphenylenediamine (PPD)

**DOI:** 10.7759/cureus.101576

**Published:** 2026-01-15

**Authors:** Sushantika Sushantika, Soumya Nanda, Jyoti Sethi, Ravi H Phulware

**Affiliations:** 1 Dermatology, All India Institute of Medical Sciences, Rishikesh, Rishikesh, IND; 2 Pathology, All India Institute of Medical Sciences, Rishikesh, Rishikesh, IND

**Keywords:** allergic contact dermatitis (acd), allergy, hairdye, paraphenylene-diamine (ppd), symmetrical drug-related intertriginous and flexural exanthema (sdrife)

## Abstract

Paraphenylenediamine (PPD) is an aromatic amine that was first manufactured in Germany by Hofmann in 1833. It is primarily employed for its antioxidant and staining properties and is found in rubber goods, printer ink, photographic supplies, textiles and footwear, henna tattoos, and hair dyes. As it is a prehapten that can cross the skin barrier easily, it can be detected in plasma, urine, and faeces after application of hair dye within half an hour. The molecular properties of PPD allow hair dye to effectively permeate the skin, resulting in sensitisation and the eventual development of allergic contact dermatitis. Redness, pain, pruritus, dryness, prickling sensation, burning or tingling sensation, and discomfort are some of the symptoms of hair dye dermatitis. Clinically, allergic reactions to PPD may vary from localised lesions, such as eyelid eczema, nummular eczema, or hand eczema, to disseminated forms, such as airborne contact dermatitis, prurigo-nodularis-like, or erythroderma-like. Systemic contact dermatitis (SCD) is a severe, delayed immune-mediated reaction (type IV hypersensitivity reaction) to PPD, a common ingredient in hair dyes and temporary "black henna" tattoos, causing widespread eczema, intense itching, redness, swelling, and sometimes even asthma-like symptoms or lymph node swelling, occurring far beyond the direct contact area after initial sensitization, and requires strict avoidance of PPD in all forms. This is a case of a woman who presented with symmetrical drug-related intertriginous and flexural exanthema (SDRIFE)-like features, a type of SCD post hairdye application, but without any history of drug intake that is relevant for Baboon syndrome (synonymous with SDRIFE).

## Introduction

The skin condition referred to as systemic contact dermatitis (SCD) occurs when a person who has been sensitized to an allergen through the skin subsequently reacts to the same or a cross-reacting allergen through the systemic route (oral, intravenous, intramuscular, inhalational, transmucosal, or transcutaneous) [1]. Common causes include drugs, metals, aromatic substances, plants, and herbal products, among others. While symmetrical drug-related intertriginous and flexural exanthema (SDRIFE) is often the most recognizable form, SCD can manifest in various presentations, including dermatitis appearing at sites previously exposed to the allergen, such as where dermatitis occurred before (recall reaction), or where previous patch tests were positive. Dyshidrotic hand eczema, dermatitis in flexural areas, exanthematous rash, erythroderma, and vasculitis-like lesions can also occur [2].

There are numerous case reports documenting SCD from drugs, yet there are hardly any reports from hair dyes. Herein, we report a challenging case of SCD who presented with a morphological appearance of SDRIFE or Baboon syndrome, maculopapular erythematous exanthem in intertriginous folds, face, arms, and trunk after contact with hair dye containing PPD.

## Case presentation

**Figure 1:**
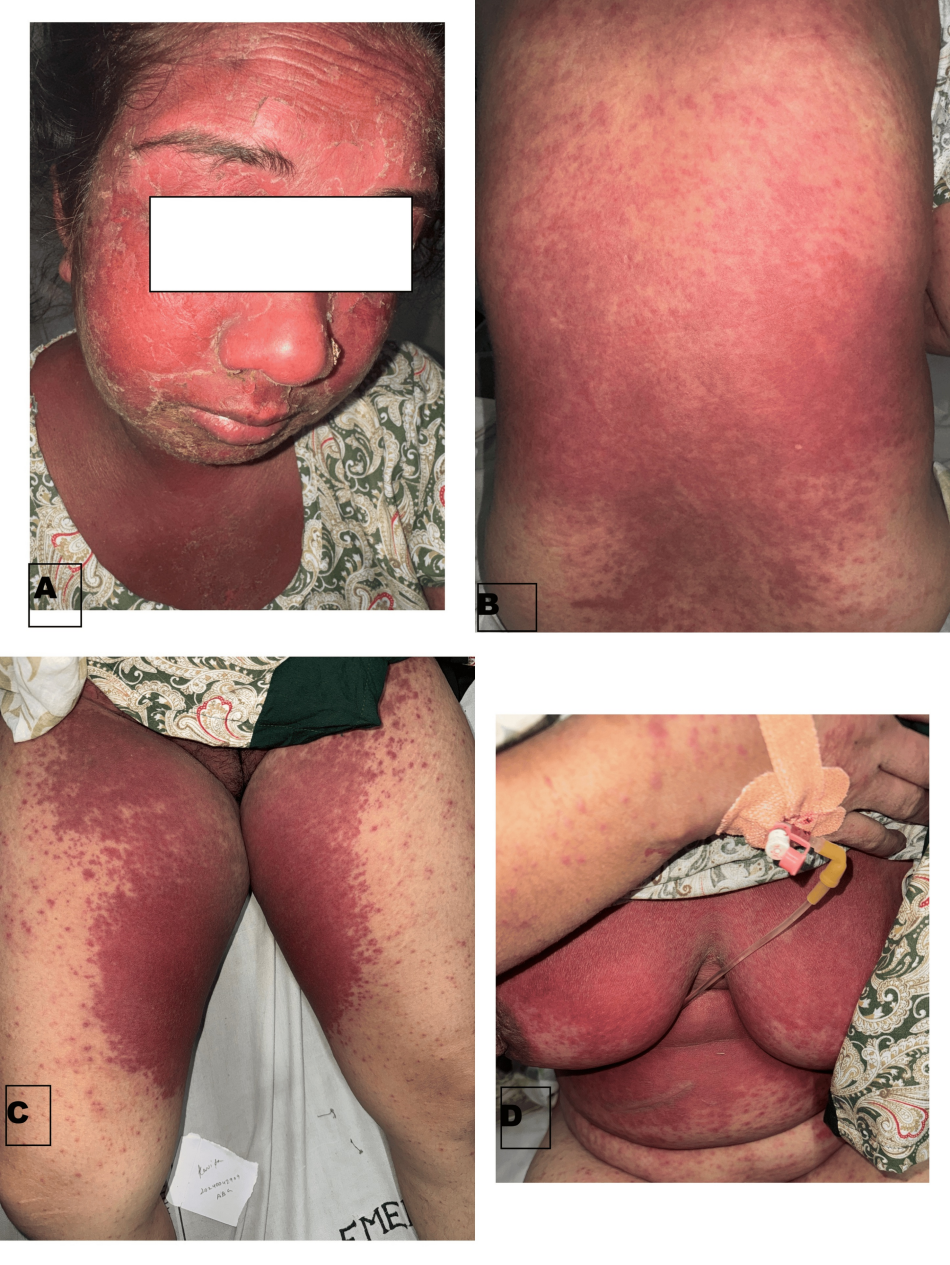
Clinical presentation of the case (a) generalized erythema of the whole face with overlying scaling and peeling. (b) Maculopapular lesions involving the back. (c) Bright red erythema over bilateral inguinal folds. (d) Similar erythematous rash over chest area.

**Figure 2:**
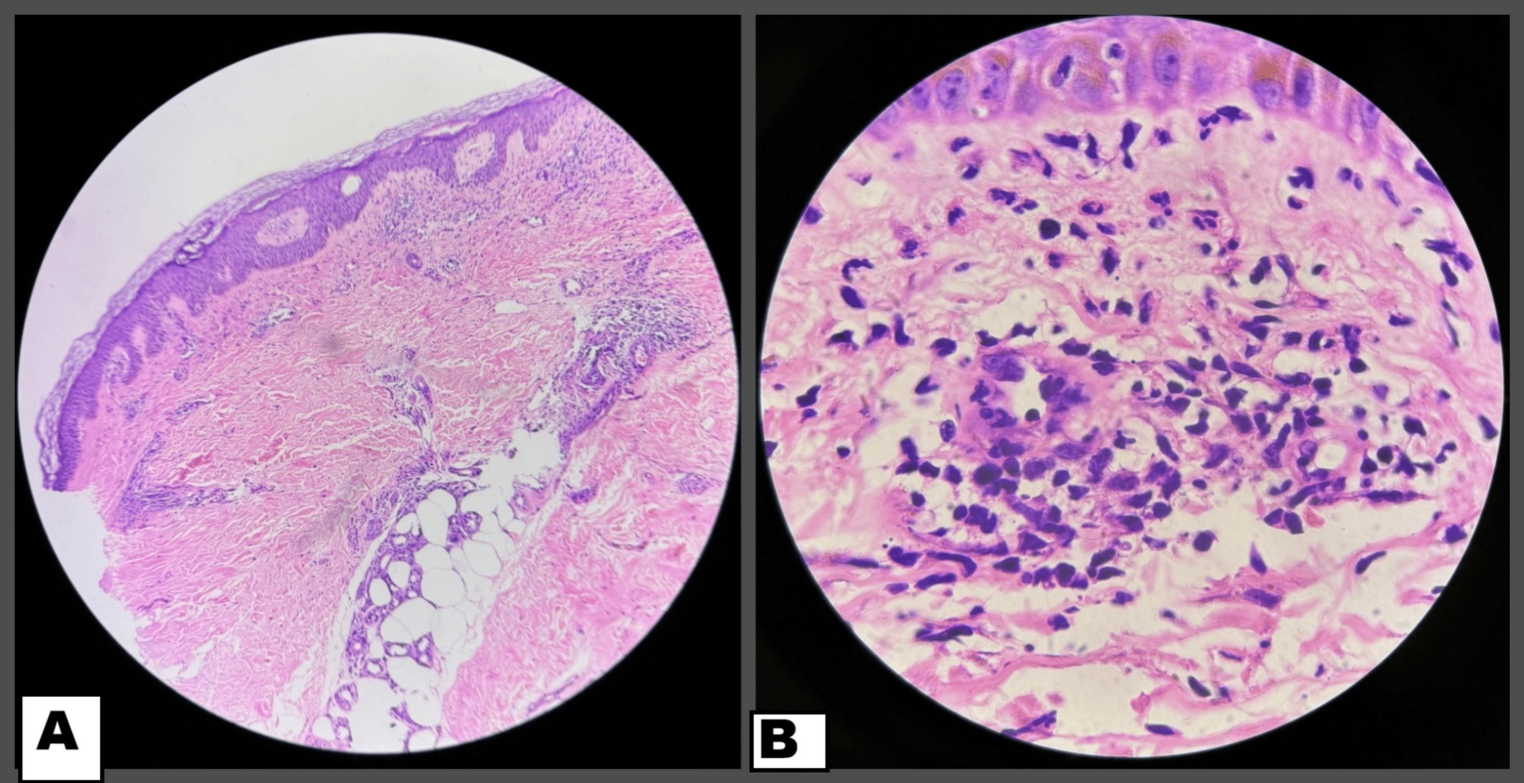
(a) Hematoxylin- and eosin-stained section (4x) showing spongiosis, with dilated capillaries and mild perivascular inflammation in the dermis, suggestive of contact dermatitis. (b) High power view of H&E (40x) showing perivascular neutrophils in the dermis.

A middle-aged female presented with itchy erythematous, blanchable maculopapules, coalescing at multiple places on her face, neck, and major skin folds, including infra-mammary, gluteal folds, axillary folds, and trunk symmetrically for two days, along with facial edema. The patient had applied black henna (hair dye containing PPD) on the scalp for coloring hair two days back, which was followed 20 minutes later by itching on her face and neck and appearance of lesions over the course of the next day (Figure 1). A history of similar pruritic lesions limited to the hairline upon black henna application in the past was also present. She was otherwise healthy with no systemic complaints. Additionally, there was no history of any drug intake or prior infection before the appearance of lesions. Differentials of drug reaction with eosinophilia and systemic symptoms (DRESS) and systemic contact dermatitis (SCD) were considered, and a thorough workup revealed leukocytosis, raised D-dimer, and a mild rise in transaminases on admission. A punch biopsy was taken from the back, and histopathological examination revealed features of spongiosis, periappendageal inflammation, along with a few dilated capillaries in the superficial dermis. No necrosis or increase in eosinophils was seen (Figure 2). With clinicopathological correlation, a diagnosis of SCD was kept, and the patient was started on systemic corticosteroids, antihistamines, and topical emollients. Following this, she showed marked improvement within the next three to four days with a gradual reduction in erythema over the next few days and complete resolution in a month.

**Figure 3:**
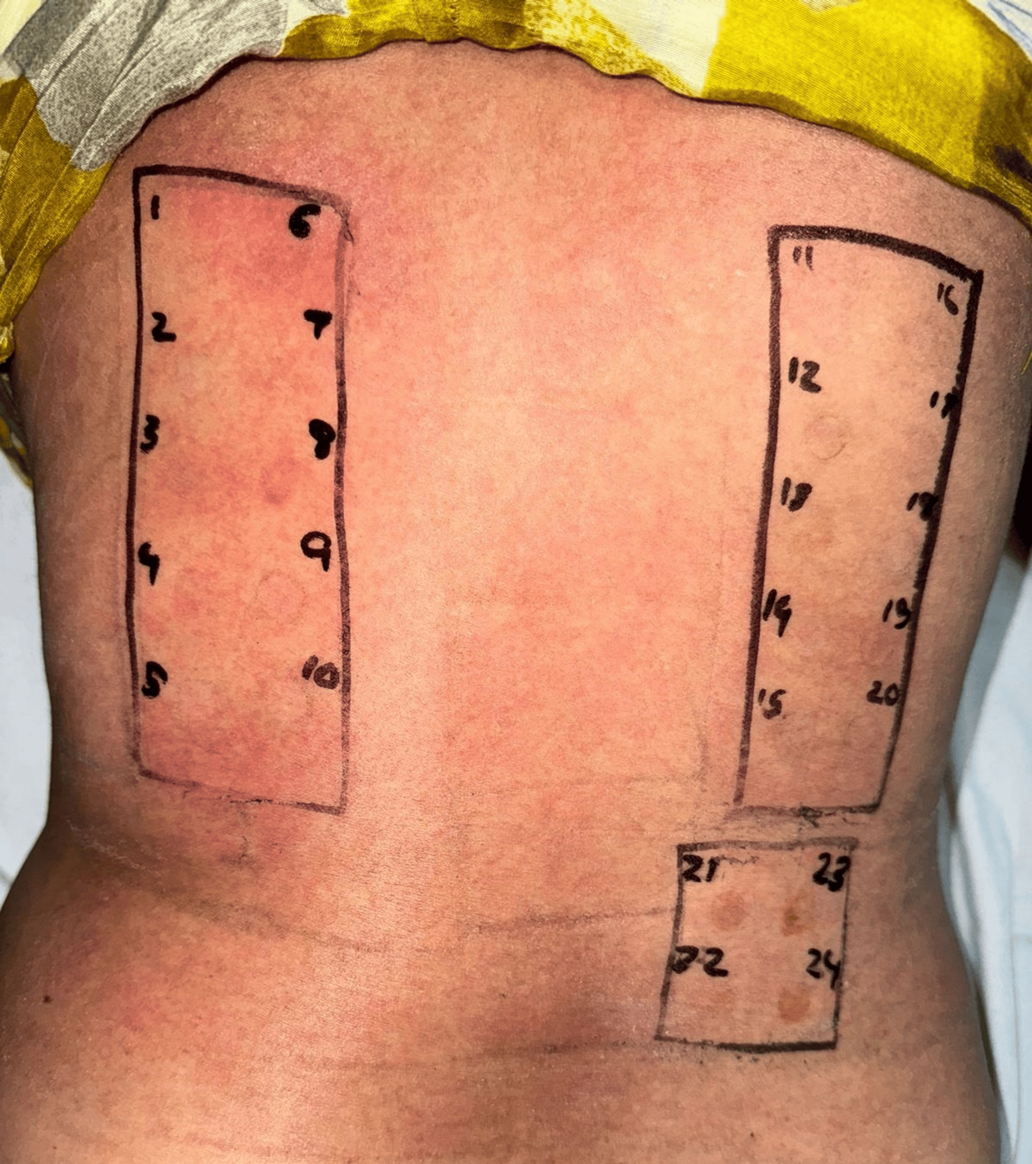
Patch testing was performed with patient's hairdye (as is) on the left side of the back and Indian standard series on the right side of the back showing flare of erythema and pruritus on the back, along with an angry back phenomenon after 48 hours.

Following resolution of symptoms in two weeks, patch testing was performed according to the recommendations of the Contact and Occupational Contact Dermatoses Forum of India (CODFI) with Indian Standard Series (ISS) that contains PPD as a standard allergen and the patient’s hair dye, as seen in Figure 3. Preparations were applied on non-lesional skin over the back with Finn chambers. A flare of erythema and pruritus on the face and body was noted, along with an angry back phenomenon on day two and day four of reading, depicting a strong positive reaction to the allergen. Following this, she was given topical corticosteroids, along with anti-histamines, with improvement in her symptoms in one week. The patient was advised to refrain from future hair dye application and, in subsequent follow-ups, showed a marked response with no recurrence of lesions.

## Discussion

When someone is cutaneously exposed to an allergen and then reacts to that same allergen or a cross-reacting allergen through a different route (oral, intravenous, transdermal, etc.), the disease is known as SCD. Although the precise etiology of this illness is not yet clarified, type 4 and probably type 3 hypersensitivity reactions are supposedly involved [3].

As SCD can be subcategorized using terminology such as Baboon syndrome, allergic contact dermatitis syndrome (ACDS) and its four clinical stages, and SDRIFE, which have multiple similarities, nomenclature is very difficult per se [4].

Systemic exposure to an allergen in a sensitized patient with subsequent development of a cutaneous delayed hypersensitivity reaction is termed SCD. Unlike localized contact dermatitis confined to the area of direct contact, SCD involves a more widespread inflammatory response throughout the body following systemic exposure to the allergen. It can be challenging to diagnose and manage due to its diverse clinical presentations and potential severity.

Baboon syndrome, one of the most well-known presentations of SCD, typically localizes in the gluteal and intertriginous areas. Herein, there was additional involvement of the face and trunk [5]. To the best of our knowledge, no reports are available on SCD due to hair dyes. It has always been reported with systemic intake of drugs or any cross-reacting agent with a prior allergen.

Clinically, SCD can have a myriad of presentations ranging from eczematous eruptions to even urticaria and anaphylaxis in severe cases. In addition, systemic features such as fever, headache, and malaise have been reported.

Diagnosis involves a combination of clinical history, physical examination, and patch testing. However, interpreting patch test results can be challenging, and false negatives can occur if the allergen concentration or application technique is inadequate.

This case underscores the clinical overlap between SDRIFE and SCD, where systemic allergen exposure elicits a delayed hypersensitivity reaction. Hair dye components (e.g., para-phenylenediamine) are notorious for allergic reactions but rarely present with SDRIFE-like features, which makes it worth sharing.

## Conclusions

SCD to hair dyes represents a complex allergic reaction that requires a high degree of suspicion. Continued research into allergen identification, diagnostic techniques, and alternative hair dye formulations can improve outcomes. Through this case, we wish to highlight the importance of a high degree of clinical suspicion, even in non-specific presentations of allergic rashes, for early diagnosis and proper management.

## References

[1] Veien NK (2011). Systemic contact dermatitis. Int J Dermatol.

[2] Winnicki M, Shear NH (2011). A systematic approach to systemic contact dermatitis and symmetric drug-related intertriginous and flexural exanthema (SDRIFE): a closer look at these conditions and an approach to intertriginous eruptions. Am J Clin Dermatol.

[3] Aquino M, Rosner G (2019). Systemic contact dermatitis. Clin Rev Allergy Immunol.

[4] Harbaoui S, Syed HA (2025). Symmetrical drug-related intertriginous and flexural exanthema. StatPearls.

[5] Häusermann P, Harr T, Bircher AJ (2004). Baboon syndrome resulting from systemic drugs: is there strife between SDRIFE and allergic contact dermatitis syndrome?. Contact Dermatitis.

